# Supplementary material for: Modeling glioblastoma heterogeneity as a dynamic network of cell states
Source: Mol Syst Biol. 2021 Sep 16;17(9):e10105. doi: 10.15252/msb.202010105 (PMC8444284; doi:10.15252/msb.202010105)
Supplement: Supplementary file 5 — Source Data for Figure 3 [file MSB-17-e10105-s001.zip › Figure3A_sourcedata/GSEA_3065/hallmarks_state4.GseaPreranked.1623416296068/HALLMARK_APICAL_SURFACE.html]

Details for gene set HALLMARK\_APICAL\_SURFACE[GSEA]

|  || Dataset | state4 |
| Phenotype | NoPhenotypeAvailable |
| Upregulated in class | na\_neg |
| GeneSet | HALLMARK\_APICAL\_SURFACE |
| Enrichment Score (ES) | -0.5231042 |
| Normalized Enrichment Score (NES) | -1.3304534 |
| Nominal p-value | 0.13322368 |
| FDR q-value | 0.16291428 |
| FWER p-Value | 0.939 |
Table: GSEA Results Summary

  

Fig 1: Enrichment plot: HALLMARK\_APICAL\_SURFACE      
 Profile of the Running ES Score & Positions of GeneSet Members on the Rank Ordered List

  

| PROBE | GENE SYMBOL | GENE\_TITLE | RANK IN GENE LIST | RANK METRIC SCORE | RUNNING ES | CORE ENRICHMENT || 1 | HSPB1 |  |  | 180 | 0.211 | 0.0846 | No |
| 2 | GSTM3 |  |  | 645 | 0.121 | 0.0752 | No |
| 3 | FLOT2 |  |  | 1569 | 0.059 | -0.0407 | No |
| 4 | TMEM8B |  |  | 2480 | 0.035 | -0.1674 | No |
| 5 | NCOA6 |  |  | 3435 | 0.014 | -0.3125 | No |
| 6 | GAS1 |  |  | 3553 | 0.011 | -0.3255 | No |
| 7 | SULF2 |  |  | 4041 | -0.012 | -0.3971 | No |
| 8 | CX3CL1 |  |  | 4209 | -0.023 | -0.4117 | No |
| 9 | SHROOM2 |  |  | 4358 | -0.033 | -0.4179 | No |
| 10 | ADAM10 |  |  | 4520 | -0.046 | -0.4192 | No |
| 11 | B4GALT1 |  |  | 5170 | -0.099 | -0.4700 | Yes |
| 12 | PLAUR |  |  | 5171 | -0.099 | -0.4169 | Yes |
| 13 | APP |  |  | 5192 | -0.101 | -0.3661 | Yes |
| 14 | THY1 |  |  | 5325 | -0.111 | -0.3275 | Yes |
| 15 | SRPX |  |  | 5662 | -0.154 | -0.2988 | Yes |
| 16 | DCBLD2 |  |  | 6104 | -0.300 | -0.2083 | Yes |
| 17 | BRCA1 |  |  | 6197 | -0.435 | 0.0106 | Yes |
Table: GSEA details [plain text format]

  

Fig 2: HALLMARK\_APICAL\_SURFACE: Random ES distribution      
 Gene set null distribution of ES for **HALLMARK\_APICAL\_SURFACE**
